# Large-scale mutational analysis in the *EXT1* and *EXT2* genes for Japanese patients with multiple osteochondromas

**DOI:** 10.1186/s12863-016-0359-4

**Published:** 2016-03-09

**Authors:** Daichi Ishimaru, Masanori Gotoh, Shinichiro Takayama, Rika Kosaki, Yoshihiro Matsumoto, Hisashi Narimatsu, Takashi Sato, Koji Kimata, Haruhiko Akiyama, Katsuji Shimizu, Kazu Matsumoto

**Affiliations:** Department of Orthopaedic Surgery, Gifu University, Graduate School of Medicine, 1-1, Yanagido, Gifu, 501-1194 Japan; Research Center for Medical Glycoscience (RCMG), National Institute of Advanced Industrial Science and Technology (AIST), Tsukuba, Japan; Department of Orthopedic Surgery, National Research Institute for Child Health and Development, Tokyo, Japan; Division of Medical Genetics, National Center for Child Health and Development, Tokyo, Japan; Department of Orthopaedic Surgery, Graduate School of Medical Sciences, Kyushu University, Fukuoka, Japan; Advanced Medical Research Center, Aichi Medical University, Nagakute, Aichi Japan; Spine Center, Gifu Municipal Hospital, Gifu, Japan

**Keywords:** Multiple hereditary exostoses, *EXT1*, *EXT2*, Mutational analysis

## Abstract

**Background:**

Multiple osteochondroma (MO) is an autosomal dominant skeletal disorder characterized by the formation of multiple osteochondromas, and exostosin-1 (*EXT1*) and exostosin-2 (*EXT2*) are major causative genes in MO. In this study, we evaluated the genetic backgrounds and mutational patterns in Japanese families with MO.

**Results:**

We evaluated 112 patients in 71 families with MO. Genomic DNA was isolated from peripheral blood leucocytes. The exons and exon/intron junctions of *EXT1* and *EXT2* were directly sequenced after PCR amplification. Fifty-two mutations in 47 families with MO in either *EXT1* or *EXT2*, and 42.3 % (22/52) of mutations were novel mutations. Twenty-nine families (40.8 %) had mutations in *EXT1*, and 15 families (21.1 %) had mutations in *EXT2*. Interestingly, three families (4.2 %) had mutations in both *EXT1* and *EXT2*. Twenty-four families (33.8 %) did not exhibit mutations in either *EXT1* or *EXT2*. With regard to the types of mutations identified, 59.6 % of mutations were inactivating mutations, and 38.5 % of mutations were missense mutations.

**Conclusions:**

We found that the prevalence of *EXT1* mutations was greater than that of *EXT2* mutations in Japanese MO families. Additionally, we identified 22 novel *EXT1* and *EXT2* mutations in this Japanese MO cohort. This study represents the variety of genotype in MO.

**Electronic supplementary material:**

The online version of this article (doi:10.1186/s12863-016-0359-4) contains supplementary material, which is available to authorized users.

## Background

Multiple osteochondromas (MO) is a relatively rare autosomal dominant skeletal disorder characterized by the formation of multiple osteochondromas and skeletal deformities, including limb length discrepancy, bowing deformities of the forearms, valgus deformity of the lower extremities, and scoliosis [1–3]. In Western countries, the prevalence of MO in the general population is one in every 50,000 individuals, and men tend to be affected more frequently than women [4, 5]. Osteochondroma is a benign bone tumor exhibiting cartilage-capped bone growth that typically originates from the metaphysis of long bones or surface of flat bones. Patients commonly feel pain or irritation of the tissues due to osteochondroma, and some patients may undergo multiple surgeries during their life in an attempt to relieve the symptoms of this disorder [6]. Malignant transformation of osteochondroma toward chondrosarcoma is a serious complication in MO and occurs in 0.38–7.0 % of patients [6–9].

The exostosin-1 (*EXT1*) and exostosin-2 (*EXT2*) genes, which encode heparin sulfate glycosyltransferases, are major causative genes in MO [10, 11]. *EXT1* is located on chromosome 8q23-q24 [12], and *EXT2* is located on chromosome 11p11-p12 [13]; these genes are essential for heparan sulfate chain elongation. Approximately 90 % of patients with MO harbor *EXT1* or *EXT2* germline mutations; however, the genetic background of patients with MO is heterogeneous. Several reports have described mutational variations and novel mutations in *EXT1* and *EXT2* genes in patients with MO in several different countries [14–17], and the distribution of mutations in the *EXT1* and *EXT2* genes has been shown to vary. For example, in Spanish patients with MO, 74 % had mutations in *EXT1*, and 21 % had mutations in *EXT2* [14]. In contrast, in Polish patients with MO, 54.6 % had mutations in *EXT1* and 30.3 % had mutations in *EXT2* [15]. Most of these mutations are inactivating mutations, including nonsense, frameshift, and splice-site mutations [18]. However, only one study has described variations in genotypes for Japanese patients with MO [16].

Therefore, in this study, we sought to determine genetic backgrounds and mutational patterns in 71 Japanese families with MO; this report describes the genetic diagnostic results of the largest Japanese cohort of MO patients presented to date and identified several novel mutations in the *EXT1* and *EXT2* genes in MO.

## Results

### Identification of 22 novel genetic lesions

In this study, all exons and intron/exon junctions in *EXT1* and *EXT2* were sequenced in 112 patients with MO from 71 families. Eighty (71.4 %) patients harbored 52 mutations in either *EXT1* or *EXT2*. All *EXT1* and *EXT2* mutations are shown in Table 1. Twenty-nine families (40.8 %) had mutations in *EXT1*, and 15 families (21.1 %) had mutations in *EXT2*. Interestingly, three families (4.2 %) had mutations in both *EXT1* and *EXT2*. Twenty-four families (33.8 %) did not have mutations in *EXT1* or *EXT2*. The distribution of mutations was as follows: 40.4 % (21/52) of patients had missense mutations, 30.8 % (16/52) of patients had frameshift mutations, 21.2 % (11/52) of patients had nonsense mutations, 5.8 % (3/52) of patients had splicing mutations, and 1.9 % (1/52) of patients had insertions. Of all 52 mutations, 22 mutations (42.3 %) were novel mutations that had not been registered in the Multiple Osteochondroma Mutation Database (MOdb) (http://medgen.ua.ac.be/LOVDv.2.0/home.php) [18]. Of these mutations, 17 mutations (77.3 %) were identified in the *EXT1* gene, while five mutations (22.7 %) were found in the *EXT2* gene. The distribution of novel mutations was as follows: 27.3 % (6/22) of patients had missense mutations, 31.8 % (7/22) of patients had frameshift mutations, 22.7 % (5/22) of patients had nonsense mutations, 9.1 % (2/22) of patients had splicing mutations, and 4.5 % (1/22) of patients had insertions.

**Table 1:** *Ext1* and *Ext2* mutations in Japanese MO families

Familiy number	The number of participants	Gene	The number of the exon	Mutation	Amino acid change	Nucleotide change	Novel/Reported	Familial/Sporadic
MO-1	4	EXT2	Exon6	Missense	p.C339F	c.1016G > T	R	F
MO-2	4	EXT1	Exon6	Frame shift	p.T488fs	c.1462AΔ1nt	N	F
MO-3	3	EXT1	Exon6	Frame shift	p.T490fs	c.1469TΔ1nt	R	F
MO-4	2	EXT1	Exon8	Frame shift	p.F550fs	c.T1650Δ1nt	N	F
MO-5	1	^a^-	-	-	-	-	-	F
MO-6	1	EXT1	Exon5	Frame shift	p.R433fs	c.A1297Δ2nt	R	F
MO-7	1	EXT2	Exon 5	Missense	p.R297H	c.890G > A	N	F
MO-8	2	EXT1	Exon1	Nonsense	p.Q27X	c.79C > T	N	F
MO-9	2	-	-	-	-	-	-	F
MO-10	1	-	-	-	-	-	-	F
MO-11	1	EXT1	Exon2	Missense	p.R341S	c.1023G > C	R	F
		EXT2	Exon2	Missense	p.R128W	c.382C > T	R	F
MO-12	2	-	-	-	-	-	-	F
MO-13	1	EXT2	Exon8	Nonsense	p.W429X	c.1286G > A	R	F
MO-14	1	EXT1	Exon6	Frame shift	p.L490fs	c.1469TΔ1nt	R	F
MO-15	1	-	-	-	-	-	-	F
MO-16	1	EXT2	Exon3	Nonsense	p.R182X	c.544C > T	R	F
MO-17	2	-	-	-	-	-	-	F
MO-18	1	-	-	-	-	-	-	F
MO-19	1	-	-	-	-	-	-	S
MO-20	1	EXT2	Exon5	Insertion	p.V282ins	c.846A insertion	N	S
MO-21	2	EXT1	Exon2	Missense	p.R340H	c.1019G > A	R	F
MO-22	1	-	-	-	-	-	-	F
MO-23	2	-	-	-	-	-	-	F
MO-24	1	EXT2	Exon5	Missense	p.R299H	c.896G > A	R	F
MO-25	1	EXT1	Exon5	Frame shift	p.P466fs	c.1395del del.	R	F
		EXT1	Exon8	Missense	p.F550S	c.1649T > C	N	F
MO-26	2	-	-	-	-	-	-	F
MO-27	1	EXT2	Exon2	Frame shift	p.F30fs	c.88TΔ5nt	N	F
MO-28	1	EXT1	Exon2	Missense	p.R340L	c.1019G > T	R	F
MO-29	2	EXT1	Exon3	Missense	p.C355Y	c.1064G > A	N	F
MO-30	1	-	-	-	-	-	-	F
MO-31	2	EXT1	Exon1	Nonsense	p.W304X	c.912G > A	R	F
MO-32	3	EXT1	Exon9	Nonsense	p.W612X	c.1797G > A	R	F
MO-33	2	EXT1	Exon1	Frame shift	p.L26fs	c.78 T (Δ1nt)	N	F
MO-34	2	-	-	-	-	-	-	F
MO-35	1	EXT2	Intron7	Splicing mutation	-	c.(1173 + 1)G > A	R	F
MO-36	1	EXT2	Exon3	Nonsense	p.R182X	c.544C > T	R	F
MO-37	2	EXT1	Exon1	Nonsense	p.Q165X	c.493C > T	R	F
MO-38	1	-	-	-	-	-	-	F
MO-39	1	-	-	-	-	-	-	S
MO-40	1	EXT1	Exon2	Missense	p.R340H	c.1019G > A	R	F
MO-41	2	-	-	-	-	-	-	F
MO-43	2	EXT1	Exon2	Missense	p.R341S	c.1023G > C	R	F
MO-44	2	EXT2	Exon3	Missense	p.A202V	c.605C > T	R	F
		EXT1	Exon1	Nonsense	p.G24X	c.70G > T	N	F
MO-45	4	EXT1	Exon1	Frame shift	p.R314fs	c.941G (+2nt)	N	F
MO-46	1	EXT1	Exon1	Nonsense	p.E74X	c.220G > T	N	F
MO-47	1	EXT2	Exon2	Missense	p.L152R	c.455T > G	R	F
		EXT1	Exon1	Missense	p.Q150R	c.499A > G	N	F
MO-48	1	EXT1	Exon1	Frame shift	p.T297fs	c.888CΔ1nt	N	F
MO-49	4	-	-	-	-	-	-	F
MO-50	2	EXT2	Exon7	Nonsense	p.Y374X	c.1122C > A	N	F
MO-51	1	EXT2	Exon2	Frame shift	p.S121fs	c.361TΔ2nt	R	F
MO-52	2	EXT2	Exon4	Missense	p.D227N	c.679G > A	R	F
MO-53	1	EXT1	Exon3	Frame shift	p.M359fs	Δ8nt	N	F
MO-54	1	-	-	-	-	-	-	F
MO-55	1	-	-	-	-	-	-	F
MO-56	1	EXT1	Exon10	Nonsense	p.Q685X	c.2053C > T	R	F
MO-57	2	EXT1	Exon1	Frame shift	p.K321fs	c.960GΔ1nt	N	F
MO-58	1	EXT2	Exon6	Missense	p.P341T	c.1021C > A	N	F
MO-59	1	EXT1	Exon1	Missense	p.I221V	c.661A > G	N	F
		EXT1	Exon5	Frame shift	p.P466fs	c.1395TΔ1nt	R	F
MO-60	2	EXT1	Exon2	Missense	p.R340H	c.1019G > A	R	F
MO-61	2	EXT1	Exon1	Frame shift	p.K218fs	Δ14nt	R	F
MO-65	1	-	-	-	-	-	-	F
MO-66	1	EXT1	Exon1	Frame shift	p.K218fs	Δ18nt	R	F
MO-67	1	-	-	-	-	-	-	S
MO-68	1	-	-	-	-	-	-	F
MO-70	1	EXT1	IVS9	Splicing mutation		intron/exon10 G > A	N	F
MO-72	2	EXT1	Exon1	Nonsense	p.E139X	c.415G > T	N	F
MO-73	1	EXT2	Exon5	Missense	p.R299H	c.896G > A	R	F
MO-74	3	-	-	-	-	-	-	F
MO-75	2	EXT2	Exon4	Missense	p.D227N	c.679G > A	R	F
MO-76	1	-	-	-	-	-	-	F
MO-77	1	EXT1	IVS5	Splicing mutation		exon5/intron G > T	N	F

^a^- indicates no mutations detected

### Characteristic genome mutations in five families with MO

**Fig. 1:**
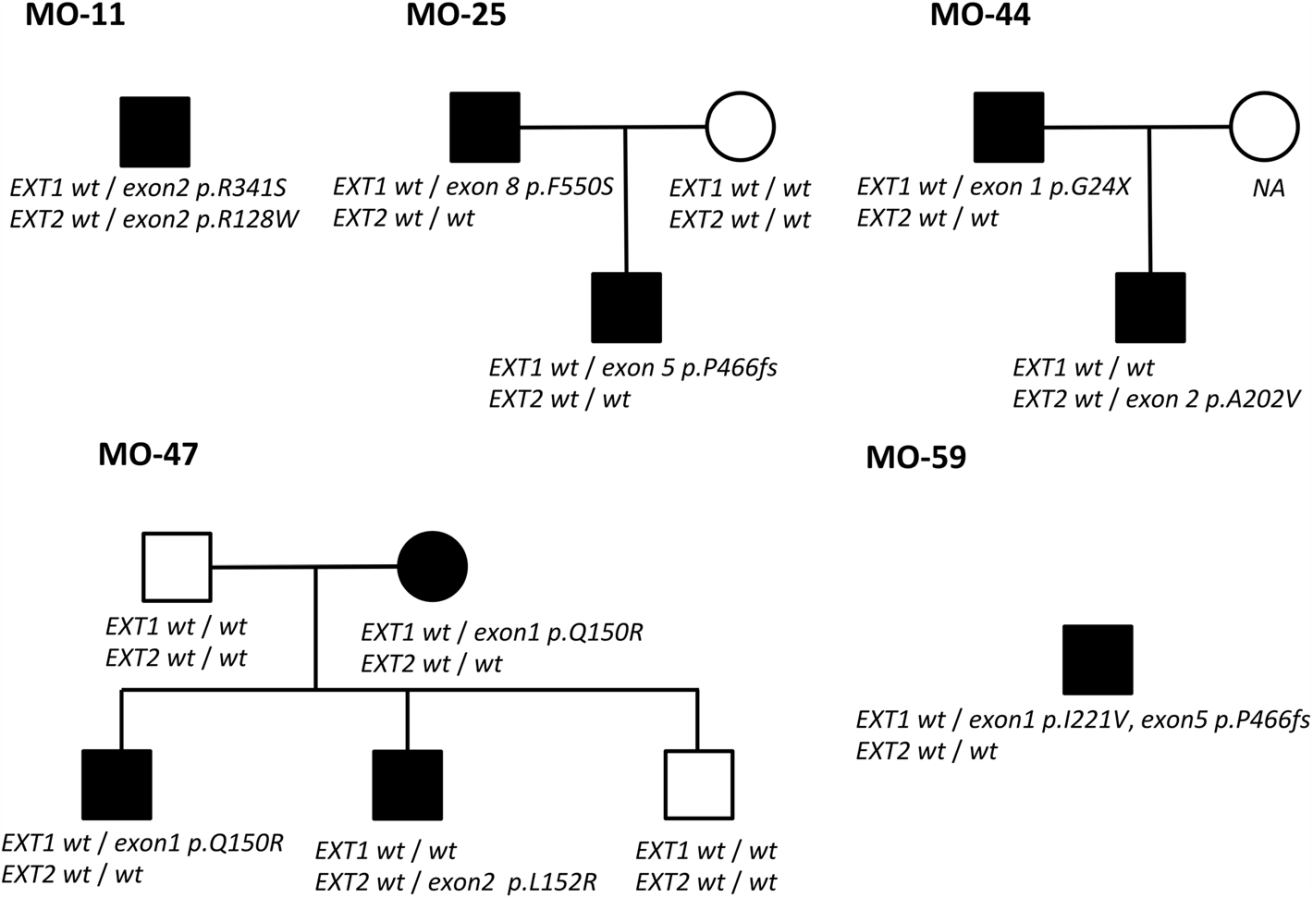
Characteristic mutations and hereditary types in Japanese families with MO. *Black mark* represents patient with MO. *White mark* represents healthy person. NA: DNA not available. Written informed consents to publish were obtained from each participants described in this figure before study participation

Interestingly, five families with MO showed unique genotypes (MO-11, -25, -44, -47, and -59) as illustrated in Fig. 1. In the family with MO-11, one MO patient harbored missense mutations in both exon 2 of *EXT1* and exon 2 of *EXT2*. Moreover, parents and children in the families with MO-25, -44, and -47 showed different genotypes. In the family with MO-59, a patient had a double missense mutation in *EXT1*.

## Discussion

In this study, we evaluated the presence and features of *EXT1* and *EXT2* mutations in Japanese families with MO. Our data demonstrated that 29 families (40.8 %) had mutations in *EXT1*, and 15 families (21.1 %) had mutations in *EXT2*. Moreover, three families (4.2 %) had mutations in both *EXT1* and *EXT2*, and 24 families (33.8 %) did not have mutations in either *EXT1* or *EXT2*. Of the 52 mutations observed in this study, 34 mutations were identified in *EXT1*, and 18 mutations were identified in *EXT2*. Of the 52 mutations 22 novel mutations were identified. Thus, the data presented herein provides important insights into the genetic causes of MO in Japanese families.

**Fig. 2:**
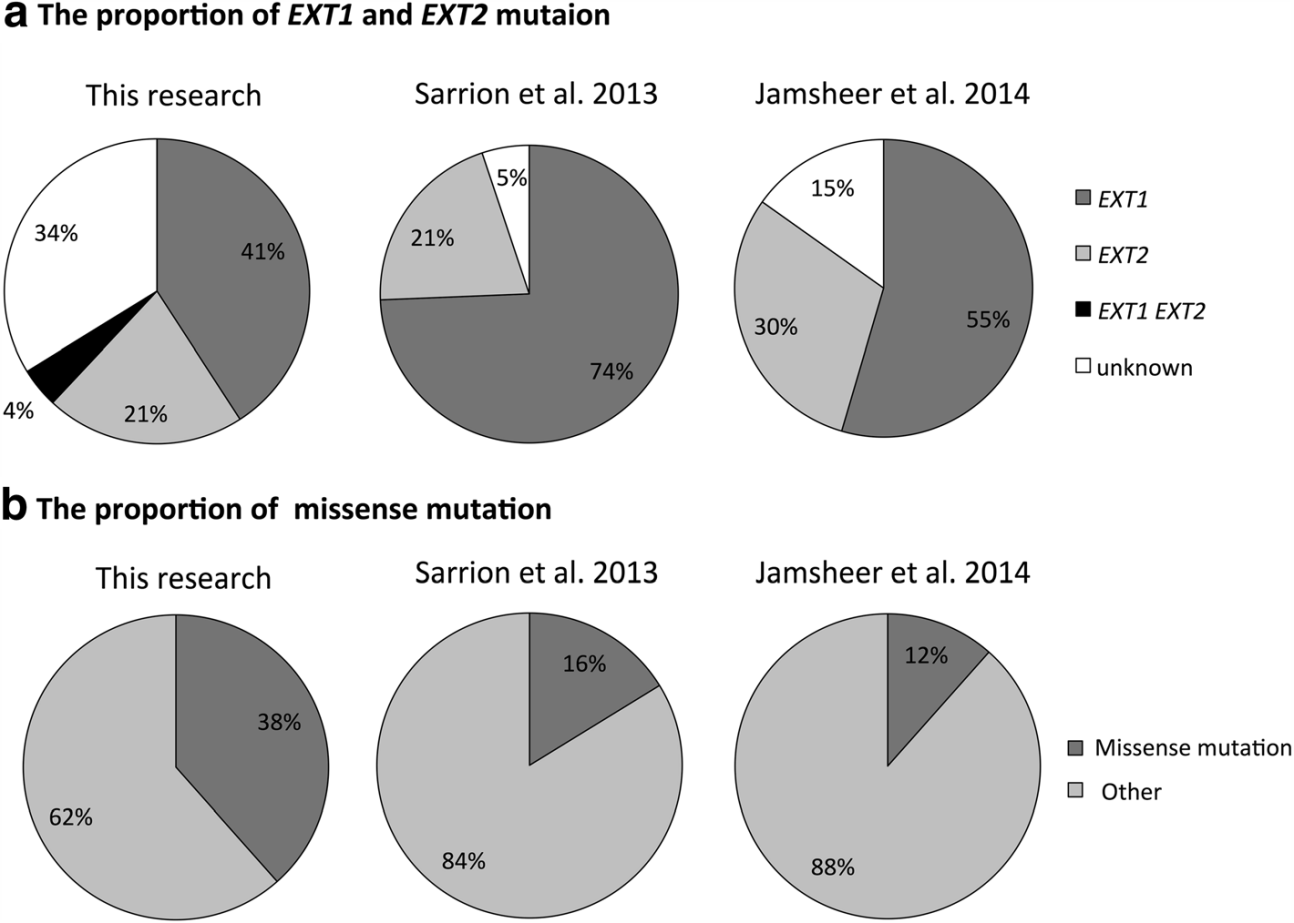
Comparison of mutation frequencies. **a** The proportions of *EXT1* and *EXT2* mutations. **b** The proportion of missense mutations

MO is an autosomal dominant disorder, and germline and heterozygous mutations conferring loss of function in the *EXT1* and *EXT2* genes are main causes of MO. Mutational variations in *EXT1* and *EXT2* are continuously being reported; as of January 2015, 432 mutations in *EXT1* and 223 mutations in *EXT2* were registered in the MOdb (http://medgen.ua.ac.be/LOVDv.2.0/home.php) [18]. Several studies have described the mutational variations in *EXT1* and *EXT2* in European countries and Asia. For example, in Spanish patients with MO, 74 % were found to have mutations in *EXT1*, and 21 % were found to have mutations in *EXT2* [14]. Additionally, in Polish patients with MO, 54.6 and 30.3 % were found to have mutations in *EXT1* and *EXT2*, respectively [15]. In an Italian cohort, 69 % of patients were found to have mutations in *EXT1*, and 27 % of patients were found to have mutations in *EXT2*. In a previous study of Japanese families with MO, 17 (40 %) of the 23 families had a mutation in *EXT1*, and six (14 %) of the 23 families had a mutation in *EXT2* [16]. In contrast, in Chinese families with MO, 13.9 and 33.3 % of the 36 families were found to have mutations in *EXT1* and *EXT2*, respectively [17]. In most studies, the prevalence of *EXT1* mutations has been reported to be higher than that of *EXT2* mutations. Similarly, in our current analysis, a greater proportion of *EXT1* mutations was observed (*EXT1*: 40.8 %, *EXT2*: 21.1 %). However, mutations in these genes were not identified in 24 families (33.8 %) with MO; this percentage was relatively high compared with that in European countries, where the proportion of patients without mutations in *EXT1* and *EXT2* has been shown to range from 4 to 24 % (Fig. 2a) [9, 14, 19, 20]. Further studies are needed to examine this finding such as MLPA assays because the families with MO harboring no mutations in this study might include deletion mutation. While the *EXT* family also includes three *EXT*-like genes (i.e., *EXTL1*, *EXTL2*, and *EXTL3*) [21–23], no reports have described the presence of gene mutations in these three *EXT*-like genes in families with MO. Therefore, further studies are needed to determine whether these three genes may be causative genes in families with MO who do not harbor mutations in *EXT1* and *EXT2* genes.

In MO, the most common mutation types in the *EXT1* and *EXT2* genes are inactivating mutations, such as frameshift, nonsense, and splice-site mutations [24]. Similarly, in data reported in MOdb from 2009, approximately 80 % of mutations in *EXT1* and 77 % of mutations in *EXT2* were found to be inactivating mutations (*EXT1*: frameshift 44 %, nonsense 24 %, splice-site 11 %; *EXT2*: frameshift 42 %, nonsense 22 %, splice-site 13 %) [18]. In Spanish patients with MO, the prevalence of inactivation mutations was reported to be 79.5 % [14]. In our study, 52 mutations were found in *EXT1* and *EXT2*, and 59.6 % (31/52) of these mutations were inactivating mutations (with 30.8, 23.1, and 5.8 % of mutations being frameshift, nonsense, and splice-site mutations, respectively). The proportion of missense mutation was approximately 38.5 %, which was relatively higher in this study than in previous reports (Fig. 2b) [14, 18]. The differences in gene mutations between patients with MO in Japan and other countries may be related to the differences in the prevalence rates of MO or the severity of skeletal abnormalities, including scoliosis, in the various countries. Further studies are required to determine the phenotype-genotype relationships in Japanese patients with MO.

In the present study, approximately 7.0 % (5/71) of families with MO showed characteristic genotypes, e.g. one patient bearing two mutations and a parent and child bearing different mutations as shown in Fig. 1. Especially, in MO-25, MO-44 and MO-47, there was one affected parent with MO and one (or two) affected child, but genotypes of child differed from that of the parents. It was reported that approximately 10 % of patients with MO exhibited de novo mutations [18]; thus, the difference of genotype between parent and child in the three families may be caused by de novo mutations although this scenario may be very unlikely. Therefore, in the future analysis, sequence or genotype might be necessary for the three families to reconfirm these results.

In MO-59, there were two mutations in one MO patient and one was a novel missense mutation. Either mutation might obtain the possibility of it being a non-pathogenic variant; however we evaluated only one genome in MO-59 families, so it is unclear whether mutation is a non-pathogenic variant.

This study has a limitation. In this study, we performed the mutational analysis in *EXT1* and *EXT2* for Japanese families with MO with using direct sequencing, but MLPA analysis has not been performed for the families with MO harboring no mutations. Thus, the families with MO harboring gene deletions might not be detected and unknown rate of *EXT1*and *EXT2* mutation potentially are high in this study. Further analysis will be necessary, and now we are planning to perform whole-genome sequencing with using next-generation sequencing technology for the families with MO harboring no mutations. In addition, in MO-25, MO-44 and MO-47, re-sequence or genotype would be performed because of unlikely hereditary form.

## Conclusions

In this study, we evaluated and characterized mutations in the *EXT1* and *EXT2* genes in 71 Japanese families with MO. A total of 52 mutations in *EXT1* and *EXT2* were identified, with 22 of these mutations being reported here for the first time. Additionally, we identified several characteristics of gene mutations in *EXT1* and *EXT2*. Approximately 60 % of Japanese families with MO had inactivating mutations in *EXT1* and *EXT2*. Interestingly, these results differed somewhat from those from other countries and represented the variety of genotype in MO. Further studies are needed to determine the reasons for these differences. This study provides important insights into our understanding of the genetic features of MO in Japanese individuals.

## Methods

### Study design and ethical approval

In this study, we performed a multicenter study at Gifu University, National Center for Child Health and Development, and Kyusyu University. Ethics Committee of Gifu University (Approval No. 22-221) approved all procedures, and all participants obtained written informed consent before any research procedures. In case of the participant under the age of 16 year old, written informed consents (child assent and parental consent) were obtained. In addition, written informed consents to publish were obtained from all patients before study participation, and in the case of the participant under the age of 16 year old, written informed consents to publish (child assent and parental consent) were obtained.

### Patients and clinical studies

From April 2010 to September 2014, patients with MO were recruited for genetic testing of the *EXT1* and *EXT2* genes. A total of 116 patients (51 women and 65 men) from 74 families with MO were recruited. Clinical diagnosis was performed based on accurate family histories and physical examinations of the patients, including palpation tests for osteochondromas or joint deformities. Ethics Committee of Gifu University approved all procedures, and all participants obtained written informed consents before all procedures.

### Mutation analysis

Genomic DNA was isolated from peripheral blood leucocytes of all patients with MO using a Wizard Genomic DNA purification kit (Promega, Madison, WI, USA). All exons and exon/intron junctions in the *EXT1* and *EXT2* genes (GenBank accession numbers NM_000127.2 and NM_207122.1) were amplified by PCR. After confirming amplification of the DNA fragments by agarose gel electrophoresis and purifying the amplified DNA fragments using a Wizard SV gel and PCR Clean-up System (Promega), the amplified DNA fragments were directly sequenced using a BigDye Terminator v1.1Cycle Sequencing kit (ABI). Sequence analyses were then performed with an ABI PRISM 3100 Genetic Analyzer (ABI). Mutations in *EXT1* and *EXT2* were evaluated by comparing DNA sequences of normal *EXT1* and *EXT2* genes with the obtained sequences using Sequencher software (Hitachi Software Engineering Co., Ltd., Tokyo, Japan). Primer sequences are shown in Additional file 1: Table S1. The detected mutations in *EXT1* and *EXT2* were examined to determine whether they had been reported previously by consulting the MOdb (http://medgen.ua.ac.be/LOVDv.2.0/home.php) [18].

In four patients of three families (MO-62, 63, 64), DNA sequence analysis could not be performed because *EXT1* and *EXT2* genes were not amplified by polymerase chain reaction (PCR). Finally, 112 patients of 71 MO families (48 women and 64 men), DNA sequence analysis was performed. All procedures were approved by Ethics Committee of Gifu University, and all participants obtained written informed consents before all procedures.

### Availability of data and materials

The Datasets used in this paper can be found at http://medgen.ua.ac.be/LOVDv.2.0/home.php [18]. All supporting data are included in the manuscript as well as additional files in the supplementary section.

## Additional file

Additional file 1: Table S1. Primer sequences for PCR amplification of the exons and exon/intron junctions of *EXT1* and *EXT2*. (DOCX 21 kb)
