# Supplementary material for: Large-scale mutational analysis in the EXT1 and EXT2 genes for Japanese patients with multiple osteochondromas
Source: BMC Genet. 2016 Mar 9;17:52. doi: 10.1186/s12863-016-0359-4 (PMC4784393; doi:10.1186/s12863-016-0359-4)
Supplement: Additional file 1: Table S1. — Primer sequences for PCR amplification of the exons and exon/intron junctions of EXT1 and EXT2. (DOCX 21 kb) [file 12863_2016_359_MOESM1_ESM.docx]

| Additional file 1: Table S1. Primer sequences | | | | |
| --- | --- | --- | --- | --- |
|  | Gene | Forward (5’→3’) |  | Reverse (5’→3’) |
| EXT1 | CpG island | CAAAGCATCTTTCTCTAGGCG |  | CTCACAAATCCCTGCATCTCTC |
|  | CpG island | GGGTTATGAATGGGCGCAGC |  |  |
|  | Exon 1 | CGAGCGCAGGAGTAAACACC |  | CGTTTTTTGGCCTGCATGTG |
|  | Exon 1 | GAGCTGAAAGTGTTGATTGG |  | GAGACTCTGCACCTTTGGATC |
|  | Exon 1 | CCTCTTTGTCCTGAGTCTGG |  | CCATCCCCCAACTTCACACC |
|  | Exon 2 | CCCCACATTCGCAATGAGTC |  | GAGAGGTGATAATGTTAAACCC |
|  | Exon 3 | CTGATTGGAACAGCTTCTGCTG |  | TGAAAGTTTGGACGGGGGCAGC |
|  | Exon 4 | GTGCATCTCTTTGTTTTACAG |  | GCTGAGAGAAGTGTATAAAGG |
|  | Exon 5 | CCTTTCCAAATATCATCAGG |  | GGCCTTTAGTTCTGTATGAC |
|  | Exon 6 | GAGCAAGGAGGAGTAATTTTC |  | ATAACAGGTAAGGAGGGCGG |
|  | Exon 7 | AAGAGGCTTTGGGTTGGAGG |  | AAGTGCCCCATGGAGAAAC |
|  | Exon 8 | GGGAGAATTGTCCTGAAAAC |  | ATCGTGCAACATGAGGTGAC |
|  | Exon 9 | TTAGTGGGGAGAAGGTAATG |  | TTCCTATTTATGCAGCAGCC |
|  | Exon 10 | GTCTCAGAAGTCCACTTGTC |  | ACGTGAGTCCTCATTACCTG |
|  | Exon 11 | CCTTGCACTTCTCTCATCATTATCC |  | GAAGAGAGAGCAGCTTGACC |
| EXT2 | Exon 1 | GCCTGAATATAAGCACCTAC |  | AAAAGCGGGCAGTCATTGTC |
|  | Exon 2 | TCAAGTGTCATTTGCCATCC |  | CCCTTCCCTTTAGTTCCCTG |
|  | Exon 3 | GGCTTGGGGATCCTTGATAG |  | ACTTCTAAATCTTCAGGAGG |
|  | Exon 4 | ACTCTGTAAACGTTAGCTGG |  | AGGACCCTACCCTGTAACTG |
|  | Exon 5 | TCAGTGGAGGTGAAGACTGG |  | CATAGGCCAAGCAGCTTTGC |
|  | Exon 6 | GTATTGCTTGGCGTCAACCC |  | GTAGTAGTTCTTGAACCAGG |
|  | Exon 7 | GGATGTTGTTTCTGCTTGTG |  | ACTCAGGCATTCAGCTCCTG |
|  | Exon 8 | CCTGGAGTTGACTATGATAG |  | TTATGCTGCCCTTATCAGGC |
|  | Exon 9 | CATGTTTGGGTTTGCTGACG |  | AAATGGAGGCATGCTGTCTC |
|  | Exon 10 | GGATACAAGCTGATTCTCCC |  | GCACACCTTTTGGACTCTAC |
|  | Exon 11 | TGGAACATCTCCAGAATCCC |  | AAGCCCTCTTGGCAGGTATG |
|  | Exon 12 | TATGAGAGAAAGCTTGTCCC |  | CCAATGTGACCGCATCAATC |
|  | Exon 13 | CATGCAACATCTCAGCTTAC |  | ACTATGGCTACCAGCTGCTG |
|  | Exon 14 | CAGACTGTGGCTACTTGAGC |  | AGTAGGTCAACCTTCCACCC |
